# Understanding the school community’s response to school closures during the H1N1 2009 influenza pandemic

**DOI:** 10.1186/1471-2458-13-344

**Published:** 2013-04-15

**Authors:** Annette Braunack-Mayer, Rebecca Tooher, Joanne E Collins, Jackie M Street, Helen Marshall

**Affiliations:** 1 School of Population Health, The University of Adelaide, Adelaide, South Australia 5005, Australia; 2 Vaccinology and Immunology Research Trials Unit, University Department of Paediatrics, Women’s and Children’s Hospital, Adelaide, South Australia 5005, Australia; 3 School of Paediatrics and Reproductive Health, The University of Adelaide, Adelaide, South Australia 5005, Australia

**Keywords:** Australia, Pandemic, H1N1, Community values, Public health response, Ethical framework

## Abstract

**Background:**

During the 2009 H1N1 influenza pandemic, Australian public health officials closed schools as a strategy to mitigate the spread of the infection. This article examines school communities’ understanding of, and participation in, school closures and the beliefs and values which underpinned school responses to the closures.

**Methods:**

We interviewed four school principals, 25 staff, 14 parents and 13 students in five schools in one Australian city which were either fully or partially closed during the 2009 H1N1 pandemic.

**Results:**

Drawing on Thompson et al’s ethical framework for pandemic planning, we show that considerable variation existed between and within schools in their attention to ethical processes and values. In all schools, health officials and school leaders were strongly committed to providing high quality care for members of the school community. There was variation in the extent to which information was shared openly and transparently, the degree to which school community members considered themselves participants in decision-making, and the responsiveness of decision-makers to the changing situation. Reservations were expressed about the need for closures and quarantine and there was a lack of understanding of the rationale for the closures. All schools displayed a strong duty of care toward those in need, although school communities had a broader view of care than that of the public health officials. Similarly, there was a clear understanding of and commitment to protect the public from harm and to demonstrate responsible stewardship.

**Conclusions:**

We conclude that school closures during an influenza pandemic represent both a challenge for public health officials and a litmus test for the level of trust in public officials, government and the school as institution. In our study, trust was the foundation upon which effective responses to the school closure were built. Trust relations within the school were the basis on which different values and beliefs were used to develop and justify the practices and strategies in response to the pandemic.

## Background

The 2009 outbreak of influenza A (H1N1) highlighted the substantial risk to health and security posed by pandemic influenza. Public health measures such as social distancing, school closures, home isolation, use of stock-piled anti-viral drugs and provision of H1N1 vaccine were important components of local, national and international responses to the pandemic. Pre-2009, pandemic planning had acknowledged the need to understand community values, beliefs and expectations if these measures were to be successfully implemented [1-3]. Yet, surprisingly little research has been conducted post-2009, to understand the experience and perspectives of those affected by implementation of such measures [4,5]. Research into the beliefs and values which underpinned community responses during the pandemic is crucial to enhance planning for future pandemics and other public health emergencies.

One domain in which community beliefs and values might be expected to be particularly important is school closures. School closures were a key non-pharmacological public health measure used to limit pandemic influenza transmission during the 2009 H1N1 Pandemic [6]. School closures are expected to slow viral transmission by limiting contact between school children, as children are thought to be particularly susceptible to influenza and highly infectious [7]. The success of the strategy relies on adherence to recommendations for home isolation and other behavioural change.

School closures pose practical and policy challenges. They transmit powerful messages to those affected, and the wider community, about the severity of the pandemic and the likely risk it poses [8]. Modelling studies [9,10] and natural experiments [11,12] suggest that school closures are only effective if invoked early, before sustained community transmission occurs. Therefore, a decision to close a school must be made under considerable uncertainty and, because information about virulence of a viral strain only becomes available during the course of the pandemic, recommendations about the need for school closures are likely to change as the pandemic progresses.

In our study we focused on school closures as a micro-instance of the impact of restrictive public health measures on the community. The aims of our study were to examine the implementation of school closures as a strategy to manage a local outbreak of a pandemic strain of influenza particularly:

• school communities’ understanding of, and participation in the closures;

• interactions between the school community and health officials; and

• the beliefs and values which underpinned community responses.

School communities in this context relates to staff, students and parents who go to or work within the school environment.

## Methods

### Recruitment and study procedure

Our study took place in one Australian city. All seven schools which had closed or partially closed during the pandemic were invited to participate in the study. In schools with whole school closures, students, parents and staff from the year level of the index case student and two or three other year levels were invited to participate. In schools with partial closures, students, families and staff in affected year levels were invited to participate. Administrative support staff such as librarians and reception staff from all schools were also invited to participate.

We included all participants who consented to be interviewed until we reached data saturation for each participant type. Data saturation occurred at the point at which no more new information was observed in the data for each participant type (school staff, parents and students). Semi-structured interviews were conducted by one of two researchers (JC or RT) between October 2009 and April 2010. Participants were asked to reflect on the impact of school closures on students and teachers and their families, precautionary measures undertaken, communication and the appropriateness of school closures and overall government response. Interviews were digitally recorded and transcribed and analysis undertaken using NVivo8 software [13].

### Study participants

Four non-government schools (one senior high school and three kindergarten-year 12 schools) and one government high school agreed to participate. We interviewed the school principal in all five schools, but one school elected not to have interviews with the school community due to time considerations. We interviewed 25 staff (excluding school principals), 14 parents, and 13 students from October 2009 to April 2010. The spread of staff, students and parents was mostly consistent across the schools, although more staff than parents and students were interviewed in all schools. Students ranged in age from 12 to 17 years and nine of the parents and students were parent-student pairs (these students and their parents were interviewed separately). Interviews typically ranged between 15–30 minutes for students, 20–40 mins for parents and teachers and 60 minutes for principals.

### Data analysis

Using thematic analysis as outlined in Braun & Clarke [14], one researcher (JC) generated initial content codes based on the topic of the conversation. For example ‘what happened when the school closed’, and ‘what were you required to do’, and ‘what did you actually do’ were content codes that related to the pandemic experience and participants’ responses [15]. A second researcher (RT) independently repeated the coding. Coding differences were resolved through discussion and thematic codes emerged based on the data and initial content codes. The codes were further refined in discussion with the whole study team. Further analysis used the lens of the ethical framework for pandemic planning proposed by Thompson et al. [1]. Framing the analysis in this way gave rise to conceptual codes such as trust, duty of care, reciprocity and protecting the public.

Thompson’s framework was developed “…to inform decision-making…[and] encourage reflection on important values, discussion and review of ethical concerns arising from a public health crisis” [1]. The framework is divided into five ethical processes, based on Daniels’ widely used “accountability for reasonableness” model [16] and ten ethical values identified from previous research and expert consultation. The processes are intended to support deliberative decision-making with the expectation that such deliberation will enhance the acceptability of actions that may need to be taken in a pandemic. The ethical values provide guidance for actual decisions; when there is tension between values, ethical processes can provide mechanisms to secure resolution. Overall, the framework is intended to work alongside the overarching goals of pandemic planning – to minimise morbidity, mortality and social disruption [17]. We have chosen to use the Thompson framework because it builds on and is consistent with other ethical frameworks, such as Carter et al. [18], Selgelid [19], Viens et al. [20] and WHO [21], but is more inclusive in terms of its categories (Table 1).

**Table 1:** Ethical processes (in Alphabetical order) - adapted by Thompson et al. 2006 from Daniels 2000

**Value**	**Description**
**Accountability**	There should be mechanisms in place to ensure that ethical decision-making is sustained throughout the crisis
**Inclusiveness**	Decisions should be made explicitly with stakeholder views in mind and there should be opportunities for stakeholders to be engaged in the decision-making process. For example, decision-making related to staff deployment should include the input of affected staff.
**Openness &Transparency**	Decisions should be publicly defensible. This means that the process by which decisions were made must be open to scrutiny and the basis upon which decisions are made should be publicly accessible to affected stakeholders. For example, there should be a communication plan developed in advance to ensure that information can be effectively disseminated to affected stakeholders and that stakeholders know where to go for needed information.
**Reasonableness**	Decisions should be based on reasons (i.e., evidence, principles, values) that stakeholders can agree are relevant to meeting health needs in a pandemic influenza crisis and they should be made by people who are credible and accountable. For example, decision-makers should provide a rationale for prioritising particular groups for antiviral medication and for limiting access to elective surgeries and other services
**Responsiveness**	There should be opportunities to revisit and revise decisions as new information emerges throughout the crisis as well as mechanisms to address disputes and complaints. For example, if elective surgeries are cancelled or postponed, there should a formal mechanism for stakeholders to voice any concerns they may have with the decision.

The study was approved by the Children, Youth and Women’s Health Service Human Research Ethics Committee and the Department of Education, South Australia. All participants and/or parents gave written informed consent before participation.

## Results

### Schools’ responses to closure

Schools complied with public health advice and requests, sometimes at great inconvenience. The unprecedented nature of the event, and the schools’ limited prior experience of anything similar, meant that they relied heavily on instruction from public health officials, whose efforts, for the most part, were praised. Schools were generally happy to accept advice from public health officials about when and how to close their schools.

Initial procedures instituted by schools at the outset of the closure were often based on generic ‘school emergency’ plans and, given the lack of prior experience, principals suggested that they had to “make things up” with guidance from government officials. Participants also indicated that the Health Department did not provide specific plans for school closures.

“Well we have a generic plan for those sort of things......, and we swung that plan into action.....But that’s more of a disaster plan and this really in a sense wasn’t disaster but it was an issue that had to be managed and contained… on an ongoing rolling basis…” [Principal, School 3]

Schools used their existing information dissemination practices, some to better effect than others. Two schools with whole school closures communicated relevant information directly through a whole school assembly. In another school, the media became aware that the school would be closing, before the principal, staff and students were informed. This caused considerable confusion: many parents called the school (some from overseas) after seeing media reports and before they were alerted by the school. Some students attended school while others did not. This confusion posed great difficulty for the principal of this particular school:

“So we were left then to split our attention three ways: we had to deal with the students who hadn’t heard the media reports and came to school anyway, and we dealt with those in exactly the same way as the whole student body had the media not been alerted before us. Then we had to deal with another tranche of students who did get the media and stayed at home and rang in and the switchboard went into meltdown. And then we dealt with the last contingent which was the media scrum on the front door”. [Principal, School 1]

Some schools used email and websites to ensure students particularly final year students, continued with schoolwork and had access to teachers during the quarantine period.

“I think it was done well. We were the first school, luckily for us we are already an on-line school. I worked at another school for 12 months while I was here, I don’t know how they would have done it because there was no student-teacher email” [Teacher, School 1]

### Interactions between schools and public health officials

The amount of interaction with public health officials varied across the stages of the pandemic but was highest in schools with whole school closures. Officials attended school closure announcements and were available to answer specific staff and student questions. Students and staff involved in whole school closures were also contacted by public health officials during their seven day quarantine to make sure they were adhering to the guidelines and their antiviral medication course and to address questions and concerns. With a few exceptions, the interactions were positive.

“we trusted fully with the Department of Health. They inspired a lot of confidence. Again, I think they were fantastic with this, and I was able to ring them and say, look, I’m struggling with this” [Principal, School 5]

Participants suggested this level of support was not available for partial closures. School principals indicated public health officials were available to them via telephone to assist but were less available to students, parents and other staff.

### Personal responses during the closures

How individuals in school communities responded was frequently dependent on the extent and nature of the information they received and how they interpreted that information. While school principals could contact public health officials, many parents, teachers and students relied on information provided by the school or through the media.

“And it wasn’t that the school said, come back to school, but I then made the decision, well if it’s been lifted and it’s on the radio…” [Parent, School 2]

Most people in the school communities adhered to quarantine but few believed that there was a severe risk. In the absence of clear instructions many invented their own rules according to their own criteria. This was predicated on their understanding of what constituted visible symptoms, the acceptable degree of contact (or lack thereof) with those who were infected, and the risk of becoming infected or infecting others.

The lack of clear instruction resulted in a range of practices. Some people stayed at home for the full seven days, had friends leave supplies on the doorstep and did not leave the house. A second group thought the school closures might be an overreaction but attempted to comply. This group adhered to quarantine for several days but, when they remained symptom-free, decided that it was safe to resume some activities such as a trips “to the shops or taking the dog for a walk”. Some parents, to avoid being seen as irresponsible, quarantined their children. However, whether the children complied with this directive is unclear as most students were home alone. One student met friends regularly although his parents believed that he remained at home. A third group saw the closure as an ineffective overreaction. This group did not quarantine other than not coming to school.

Very few people were extremely concerned that they might become infected or transmit the virus to others. Those who were most concerned were pregnant women, and those with young children.

“But he said, look, I just want to let you know because you’ve got - your partner’s pregnant and it might not be good for you to come in. But we are having a briefing as well, and explained that the Year 10s would be getting the Tamiflu and their teachers should be as well. So when I asked who the student was, and I was told it was a Year 10 student of mine that I had quite a lot of contact with over those last two days. So I spoke to my partner about it and we decided that I would go in to find out exactly what was going on, and to collect that medication, and then I would talk to her as to what we’d do. But, you know, basically we’d agreed that she and - I’ve got a two - two-and-a-bit year old son, they would move out for a week”. [Teacher, School 1]

Staff at some schools maintained close contact via email and phone and as a group determined that they were safe to re-enter the community before the quarantine period was completed. This was based, in part, on rumours circulating through these informal staff cliques that other staff members (including the principal) were already out in the community.

“Yeah. Yeah. We were all, you know, sort of either emailing or talking to each other going, well what do you think? No one’s sick. Right, okay. Reckon it’s safe? We still tried to minimise. It’s not like we’d go to massive sporting events or…” [Teacher, School 3]

In summary, the school closures exhibited several features which made the situation particularly challenging: there was clearly urgency, but no clear response plan; there was considerable uncertainty, both about disease severity and of the correct response; recommendations for the imposition, continuation and lifting of closures and home isolation changed rapidly; and the key sources of authoritative information for students, families and staff were school leadership teams, who were not infectious disease experts.

### Making meaning of school closures in a pandemic: ethical processes and values for schools, parents, students and policy makers

To build an effective response to a pandemic in the future, we will need to understand not only *what* school communities are likely to do but also *why* they respond in certain ways. Using the framework of Thompson et al. [1], we examined the ethical processes and values employed in the school communities’ responses to school closure. The framework has two parts: the first focuses on ethical processes and the second on underpinning values. Both components are important. Ethical processes can lend legitimacy to actions during a pandemic, so that stakeholders are more able to accept the difficult decisions that may need to be made. However, as Thompson et al. [1] noted, “ethical processes do not guarantee ethical outcomes” and their framework therefore also has “ten key ethical values to guide decision-making that address substantive ethical dimensions of decision-making in this context”.

Overall, we found that there was considerable variation between and within schools in their attention to ethical processes and values. Schools varied in the extent to which information was shared openly and transparently, the degree to which school community members considered themselves participants in decision-making and their thoughts about the responsiveness of decision-makers to the changing situation. In all schools there were reservations about the need for closures and quarantine and a lack of understanding of the rationale for the closures. However, in all of the schools, members of the school communities could articulate core values such as providing care to all affected by the pandemic, protecting the public from harm and promoting wise use of resources. To some degree in each school, coordination and cohesion between public health officials and community members and the protection of privacy, equity and liberty were also acknowledged and valued. In contrast, all the schools struggled with the notions of proportionality and reciprocity. In the following sections we discuss the ethical processes (Table 2) and values (Table 3) apparent in the descriptions provided by our participants.

**Table 2:** Illustrative data extracts- Thompson’s framework- ethical processes

**Ethical processes**	**Ethical decision-making or decisions should be…**	**Evidence of ethical processes in study**	**Examples - positive**	**Examples - negative**
Accountability, openness and transparency	sustained throughout the crisis and publicly defensible – processes should be open to scrutiny and reasons for decisions publicly accessible	Mixed – strong processes in some schools but not in others	“That Monday morning we had signs on all the gates to say that the school was closed to year 10 students. We had staff monitoring all the gates to make sure if a year 10 student didn’t get the message that we would send them home. Actually we didn’t have a single student turn up…, but I think having the SMS messaging system was absolutely critical. The feedback we got afterward was that instant contact with parents through SMS, through emails, through phone calls, …it was really critical”. [Principal, school 5l]	“It was clear that we were to stay home. It wasn’t clear whether people could visit us. It wasn’t clear whether our family could leave. So the people we lived with…That wasn’t clear but it was very clear that we had to stay home for the week.” [Teacher, school 3]
				“If they want them home and in quarantine from potential contact, then it really should be stated that that's what they want. Some people are just going to ignore it anyway, no matter what you do, but for people who do actually read and take notice of these things, it makes it easier to make a more informed decision like yes, I can see the point of this, I will keep them home, not let their friends come around, not take them to sport or out shopping with me sort of thing” [Parent, school 4]
Inclusiveness	made with stakeholder views in mind and stakeholders should be engaged in the decision-making process	Inclusive approaches in some schools but not in all (leading to confusion in message transmission)	. . . we were aware that through Facebook, things were going out absolutely everywhere. So we thought, we can’t stop that, what we can do is educate the [students] so that what they are sending out is informed, we ran through our student leadership groups, we ran seminars . . . to say . . . if you’re going to be talking about this, this is what you should be talking about . . . if you’re going to be talking to your mates or talking to . . . mates of mates, which happens with Twitter and Facebook or whatever, this is what you should be saying.{Principal, school 5]	“.......it became a directive that you must close those classes. … Generally the school knew that that wasn't going to stop it because everyone in the school we believed had some contact with someone else who actually knew that child, so it was either all school closure or non school closure.” [Principal, school 4]
Reasonableness	based on reasons (evidence, principles, values) and made by people who are credible and accountable	In general, perceived lack of clear rationales for closures		“It was clear, like just stop I guess interaction with one another and just stop the spread so that was really clear and that’s why I guess I definitely stayed home for a while…It wasn’t clear why we would meet altogether in the gym if that was the case so… Like there was some irony I guess in meeting altogether…” [Teacher, school 3]
				“The official line was we refer to the government website … and a lot of the information on there was quite ambiguous..... And it was really putting the onus back onto the individual to make a judgement, as was the case with me. That’s why I made the judgement that well I don’t have any symptoms, I haven’t taught that boy directly, I haven’t come in contact with that boy directly, therefore it’s highly unlikely that I would be irresponsible by going interstate. So, I did, and I was fine”. [Teacher, school 4]
				“and the real confusion lies in that the kids were told that they shouldn’t go out and they should stay fairly contained but be at home with family. But the family could all go out and they couldn’t see the logic in it…” [Teacher, school 3]
Responsiveness	revisited and revised as new information emerges	Mixed – while policy changes responded to the changing situation, some members of school communities interpreted the policy shifts as inconsistent and not responsive to local circumstances		“I think the biggest issue with school closures…was it wasn’t consistent. They changed the rules and then even after we excluded people, the initial exclusion was for a week but then we changed the rules and told them to come back after two days. So I think that you have to be consistent and there’ll still be phases. I can understand phase one, phase two, phase three or whatever phases they need because once it’s reached a certain point you realise that isn’t working and it’s probably more disruptive than it’s doing any good”. [Teacher, school 2]

**Table 3:** Illustrative data extracts- Thompson’s framework- ethical values

**Ethical values:**	**Decision-makers must strive to:**	**Evidence of ethical values in study**	**Examples - positive**	**Examples - negative**
**Values which were strongly in evidence**				
Duty to provide care	– work collaboratively with stakeholders to establish practice guidelines	Strong: All groups took duty to care seriously; schools had a broader definition of duty of care.	[Do you think that closing the school was an appropriate response…]	
			At that time, yes I do. Yes. Because I think that, again, if it had spread – [but] I mean, I teach a Year 12 class and we simply cannot afford the time to be mucking around and just staying away, et cetera… [Teacher, school 3]	
	– develop fair and accountable processes to resolve disputes			
			“Well, I guess when we knew that it wasn’t even as strong as the normal flu or whatever so that seemed kind of stupid but then there was the other argument which was; what if it turns into something really bad”. [Student, school 2]	
Protection of the public from harm	– weight the medical and moral imperative for compliance	Strong – good awareness of need to protect public from harm	“… I really thought some of the rules were totally ridiculous but I didn’t want to put others at risk … I did take the dog for a walk but I made sure I went on a route where I wasn’t going to come in contact with anyone because I can’t sit still for 24 hours…But I think it was more – like I wouldn’t have visited my brother because I didn’t want to put his kids at risk.” [Teacher, school 2]	
	– ensure public are aware of medical and moral reasons for public health measures			
	– ensure public are aware of benefits of compliance and consequences of non-compliance			
	– establish mechanisms to review decisions as public health situation changes			
			[So thinking about the fact that you stayed home for that whole week and sort of stuck to the regulations I guess, what sort of promoted you to do that? Why did you stick to the rules?]	
			“Um.... There wasn’t really any point for me going out anyway, you know. I’d rather stay home, just stick to what rules were set then…yeah, ‘cause if I did have the infection I didn’t want to spread it any more.” [Student, School 4]	
Stewardship	– consider benefits to the public good and the fair distribution of benefits and burdens	Strong- serious efforts to minimise harm and use the pandemic for good ends	“Before I left to go home that day I scanned in all of my lesson plans and all the resources for my Year 12 classes, which I was most anxious about. And so I emailed those to all of my students, and I was in phone contact, I was on email, and I set them enough work to make sure they didn’t lose any time or we didn’t fall behind in our course......I didn’t mind doing that. … And with my Year 10s, I didn’t set them as much work, and I was available through email and I gave them my home phone number”. [Teacher, school 1]	
**Values for which evidence was mixed**				
Solidarity	– ensure good, open, honest communication	Mixed: Solidarity between PH officials and schools in some situations, but not all.	“Look, Communicable Diseases were fantastic. They were probably in the same spot we were....”. [Principal, school 3l]	“Effectively the only thing we really got told was to … try not to go to public places etc . . . a few of us [were] going out to dinner on Saturday night, so we cancelled that, then found out [a school leader] went to the football that Friday night. [The school leader] said that some information had come through later that we didn't have to be as isolated. That wasn’t conveyed to staff in any way, shape or form, which I thought was pretty disgusting. . .” [Teacher, school 3]
	– open collaboration in a spirit of common purpose between institutions		“I would say as the events unfolded that the communication … from the school was outstanding and from the Health Department who were here and giving information to the kids and to parents. There were daily phone calls to find out whether [our son] had shown any symptoms and when we were at work and he was at home on his own those phone calls were still there.” [Parent, school 1]	
	– share public health information			
	– coordinate delivery of health care and deployment of human and material resources	Lack of solidarity undermined School closures		
Privacy	– disclose only private information relevant to achieve legitimate and necessary goals of public health	Mixed: good awareness of privacy issues but sometimes practices undermined this	“So, you know, we sort of, we really wanted to stress to the media that this is a human story It was about the dignity of the child that’s ill, and the dignity of all people that [have] swine flu…” [Principal, school 5]	“Oh, there was nothing subtle about it. Everyone knew that if you were the three students . . . that got the pink one [form], that meant it was really bad, and if you were the 27 kids that got the orange colour, then that meant it was really good . . . They are very attuned - the kids know . . . they pick it up straight away. . so they knew straight away…..yeah, the pink one - oh my god, I’m going to die. It was like, oh my god, that’s it; and then the bursting into tears stuff.... Because no one really knew”. [Teacher, school 4]
	– release private information only if there is no less intrusive means to protect the public.			
	– provide public education to correct misperceptions about disease transmission			
Equity	– preserve as much equity as possible between interests of those with influenza and others	Mixed: awareness of equity issues but sometimes practices undermined this	Some of the wording from the Department is very hard for families to understand. They would read something like a release and they would ring up and ask what does that mean? We would have to explain that means this. Why doesn’t it say that? It does but [not] someone whose third or fourth language is English. [Principal, school 3]	“Totally confused about the home isolation. We tried to clarify that through the web and realised how many families and parents don’t have [access to] the web”. [Principal, school 3]
	– ensure procedural fairness in decision-making			
				“… the Year 12s … they obviously blamed the Year 10s for bringing it in and … then they all started hating us… The [student]who had it … had to have a couple of days off when [they were] better because [they] just couldn’t handle people…sneering at [them]…” [Student, School 1]
Individual liberty	– ensure restriction to individual liberty are proportional to harm, necessary and relevant to protecting public good, employ least restrictive means and are applied without discrimination	Mixed: range of views about whether restrictions on liberty were justified	They said there was a real concern. They were really concerned about it. Therefore you must take it with some seriousness. [School librarian, school 1]	“..He [stayed home] pretty well but I think he went down and had a hair cut at the local hairdressers and a couple of other things. I don’t think he mixed with many other students. But he went off and did his violin lesson as he normally does....As far as I was concerned, if he was symptom free, the risk of him transferring it was no greater than anyone else in the community.” [Parent, school 2]
**Values for which there was little evidence or contrary evidence:**				
Proportionality	– use least restrictive or coercive measures to limit individual liberties or entitlements	Lacking: Most members of school communities thought the response was not proportional to the danger		“. . it would be the wrong thing to say it was a sop to the policy, but that’s what it turned out to be, because in reality we had one student who had minimal contact with a small number of students, and we shut the whole place down as a result of that.” [Principal, school 1]
	– use more coercive measures only when less restrictive measures have failed to meet public health goals			
Reciprocity	– ease burdens of stakeholders responding to public health measures	Lacking: little to no awareness of impact on individuals; stigmatisation		“… I did ring up a girlfriend and asked her to do some shopping she dropped things on the front verandah, knocked on the door, and went. [sigh] so it was a bit of a lonely time for me. When you’re on your own, to be totally isolated with no one. With no contact with anyone, except the telephone.” [School librarian, school 1]
**Key value**				
Trust	– take steps to build trust with stakeholders before a crisis	Mixed: not strongly apparent in all schools, but where displayed school closures worked well	....it was difficult when I didn’t quite understand it, but then, we trusted fully with the Department of Health. They inspired a lot of confidence. I think they were fantastic with this. [Principal, school 5]	“. . . you don’t know how much PR was involved versus actual medical necessity . . when any government agency is making a decision…they need to be seen to be handling it in a controlled way . . . for fear of being criticised in the media for it.” [Teacher, school 2]
	– ensure decision-making processes are ethical and transparent to affected stakeholders			
			“Someone got positive for swine flu in my grade. Everyone was a bit nervous but a lady came along and she told us it was alright and stuff. But yeah, the principal was saying, just everyone keep calm. [Student, school 3]	

### Ethical processes and the practices adopted during the school closures

#### Accountability, openness & transparency

According to the Thompson framework (see Table 2), transparency is essential for building and maintaining public trust in public health decisions and displaying accountability. Although all schools demonstrated competence and familiarity with communication strategies, not all communication was open and transparent. Much of the confusion, frustration and lack of adherence with home quarantine and other measures associated with the school closure stemmed from a perception amongst some members of school communities that health officials and government were not being transparent. In particular, some participants believed information about the severity of the pandemic and virulence of the virus was withheld.

#### Inclusiveness

Thompson et al. stated that ethical decision-making should be made with stakeholder views in mind and stakeholders should be included in decision-making. In our study, there is some evidence that this was accomplished effectively, with the school and health department staff working closely together to shape the information for families and its timing and format. One school also worked with student groups to enhance the quality of the information transmitted via social media.

However, stakeholders were not always included in the decision-making processes, at a whole school or individual level. In these cases, the messages that were transmitted about home quarantine and school closures seemed to be poorly understood. In particular, the lack of specific guidelines for the range of situations which presented exacerbated confusion for families.

#### Reasonableness

The government’s failure to adequately convey a clear rationale for the measures undertaken tended to reduce the credibility of information and led to widespread misinterpretation of the advice about home isolation. Misunderstanding was exacerbated when the ways in which the closures were effected seemed to be at odds with the advice being given (see Table 2). Taken together with the rapidly changing situation, these factors led students, parents and teachers to construct their own rationales and act according to their own assessment of what they should do.

#### Responsiveness

Ethical decision-making requires that decisions be revisited and revised as new information emerges. The rapidly changing instructions about school closures, from a policy of whole school closures, to partial school closures, to no school closures within a month, could certainly be seen as responsive. However, it is clear that the school communities had difficulty understanding these changes and felt that their views and interpretations were not always taken into account. The government and public health officials failed to flag in advance that changes were likely to occur and, as changes happened, failed to adequately explain why the changes were required. Thus, rather than being responsive, some members of school communities interpreted the policy shifts as inconsistent and as evidence of lack of interest in their views.

### Ethical values important during the school closures

The ethical values that were articulated by the members of these school communities (see Table 3) can be separated into four groups: values which were strongly in evidence in all settings (the duty to provide care, protection of the public from harm and stewardship); values which were adopted but variably expressed (solidarity, privacy, equity and individual liberty); values for which there was little evidence or contrary evidence (proportionality and reciprocity) and a final value, trust, which played a foundational role underpinning other values.

### Values which were strongly in evidence

#### Duty to provide care

Health officials and school leadership teams all had a strong commitment to provide high quality care for the members of the school community, although, they were driven by slightly different professional understandings of that duty. Health officials appeared to be working towards the accepted public health goals of minimising morbidity and mortality while using the least restrictive measures available to slow the spread of the infection. Schools on the other hand, had a broader notion of duty of care: they wanted to ensure the welfare of the whole school community and, at the same time, provide quality teaching and learning for students. Schools were willing to work closely and collaboratively with public health officials to ensure that they were adhering to the recommendations (to fulfil their broad obligations to protect the school community) and they needed, at the same time, to consider the educational impact on students.

#### Protection of the public from harm

Clearly, the need to protect the public from harm is at the core of the pandemic mitigation strategies. Many teachers, parents and students clearly understood this and also appreciated the need to maintaining a balance between protecting the public from the spread of H1N1 and ensuring that the school closures did not cause other unintended harms.

#### Stewardship

Schools, and some teachers in particular, were acutely aware of the possible disruption to the learning of final year high school students and they went to significant lengths to minimise this, even when teachers were themselves in home isolation. In addition, some schools took the opportunity to use the pandemic for other educational goals, including reinforcing hygiene practices such as hand washing, sneeze etiquette, and cleaning of surfaces, or focusing on pandemic literacy generally.

### Values for which evidence was mixed

#### Solidarity

There was a more mixed commitment to the value of solidarity. Where schools and public health officials worked closely together to ensure that school communities had adequate information, there was a strong sense of shared purpose. Personal contact with public health officials seemed to engender the strongest feeling of a collaborative effort to mitigate the effects of the pandemic. Personal contact also increased individual parents’ and students’ confidence in management of the closure.

However, when personal contact between health department staff and participants was more limited, this appeared to reduce the sense of working together to combat the pandemic. Solidarity was also undermined when different rules appeared to apply to different members of the school community.

#### Privacy

Schools were acutely aware of the need to try to protect the privacy of affected students. However, their efforts were hampered by rapid spread of information via social media and a significant amount of media coverage. The way information was conveyed in some schools also undermined privacy: in one school, all students were handed a note giving information about the closures; however, students who were required to adopt home isolation (because they may have been in contact with the student diagnosed with H1N1) were given a note in a different colour, clearly identifying them to other students and staff.

#### Equity

Equity (or the lack of it) was an important issue, but school communities felt they could do very little about it. For example, access to the internet was assumed, but limited, for some students and parents, and public health officials also were not prepared for the provision of information in languages other than English, or to suit varying degrees of health and general literacy. Some schools filled this gap with their own processes for managing language difficulties.

#### Individual liberty

Balancing restrictions on individual liberty with the need to protect the public from harm was a key challenge in managing the pandemic. It was clear among those affected by the school closures that some valued their individual liberty more highly than others, and this was reflected in their response to the closures, in particular their lack of acceptance of the need for home isolation.

### Values for which there was little evidence or contrary evidence

#### Proportionality

Most members of school communities felt that the response required of them was disproportionate to the danger posed to the community by the pandemic. This perception may have developed in hindsight (which our participants had). However, the lack of transparency about the rationale for actions and the rapid changes in the advice provided, without additional explanation about those changes, undermined the sense that the school closure would be effective in limiting the spread of disease.

#### Reciprocity

“Reciprocity not only requires that individuals should not be overly burdened by measures to protect public health, but also that individuals are supported in a way that allows them to fulfil their obligations” [20]. Some of our interviewees thought that the adverse impact of home isolation on them had not been taken into account. We also found that some level of stigmatisation was experienced by the initially affected student in each school and subsequently by the student’s class or year level. Sometimes the stigmatisation contained implicitly racist elements.

### Key value: trust

Thompson et al. place ‘trust’ at the end of their list of key ethical values. This is a good positioning from the perspective of the findings of our study, as trust was a key ethical value underpinning the responses, practices, and strategies used during the school closures. Where closures happened effectively and smoothly, members of the school community appeared to trust that the closure strategy was important to the health of the nation and would be effective in slowing the spread of the influenza virus [22]. They also trusted that complying with the advice of the government to close schools was in the best interests of the school community [21].

## Discussion

Despite similar challenges, there was considerable variation between and within schools in the ways in which ethical processes and values were enacted and interpreted. With respect to processes, *accountability, openness and transparency*, and *inclusiveness* were apparent in some schools, but not so clearly in others. All schools displayed a degree of *responsiveness* to the changing situation, but some members of school communities interpreted the policy and procedure shifts as inconsistent and unresponsive to local circumstances. Finally, participants across all schools thought that the ways in which the closures were managed failed the *reasonableness* test. In general, the government’s failure to adequately convey a clear rationale for the measures they were taking gave an impression that decisions were not being based on good reasons.

With respect to values, the picture was even more complex. All schools displayed a strong awareness of their *duty of care* toward their students, although members of the school communities had a broader view of care than that of the public health officials. Similarly, there was a clear understanding of and commitment to *protect the public from harm* and to demonstrate responsible *stewardship*.

There was a more mixed commitment to the values of *solidarity*, *liberty*, *privacy* and *equity*. While there were clearly shared aims and cohesion between public health officials and schools in some situations, this solidarity was undermined by lack of clear information and differences in interpretations of restrictions on movements by various members of the school communities. Similarly, although all schools were generally aware of privacy and equity issues, practices instituted in haste without the benefit of forward planning, acted to undermine these values.

Finally, in accordance with their understanding of the unreasonableness of the closures, most members of school communities considered that the response of closing the schools was in no way *proportional* to the danger posed to the community. They also thought that insufficient attention had been paid to *reciprocity*. Little account appeared to have been taken of the impact of quarantine on individuals or the stigmatization that was attached to being labelled as a ‘case’.

Underlying all of these values and processes was the value of *trust*. Trust was the foundation upon which effective responses to the school closure were built. Trust relations were the basis on which different values and beliefs were used to develop and subsequently, to justify the practices and strategies that were undertaken. Simplistically put, a school closure seemed to work well when there were high levels of trust between all players. In an environment of trust, the school community could put in place processes that were ethical and which were an expression of the school’s underlying values. An environment of trust turned the pandemic into an opportune learning experience. This was well expressed by one of the principals:

Look in some respects, yes, our students weren’t there for a week, but I think what we learnt as a community from this far outweighed what the [students] would learn in a week from that. I thought they learnt a lot about the Human Swine Flu, about a lot of things from this, about resiliency and about community, about compassion. So, you know, they’re our four strategic values, we’ve got faith, excellence, community, compassion. I think we learnt something about all those four areas. (Principal, School 5)

The extent to which public health officials are able to harness the existing trust relationships between school communities and their school as an institution may itself depend on the stability and endurance of those relations and the school’s efficacy in managing its day to day business [23]. Thus, in schools where trust relations are already fractured or weak, or where the day-to-day business of managing the school is problematic, public health emergency managers could not rely on those trust relations to build trust with the community indirectly. In other words, if trust within the school community is already low, then it cannot be used as a resource to mobilise community adherence with the school closure strategy, and in fact may be a barrier to action.

## Conclusions

The findings reported in this study concerning both what schools did, and the values which underpinned their actions, are important for understanding broader community responses to a pandemic and preparing for future infectious disease outbreaks. There are, of course, limitations inherent in this study. It was undertaken in one Australian city; thus, to some degree, relations between school communities and public health officials reflect the quite specific circumstances of this city. In addition, not all opinions will have been captured in the limited sample size, but we believe our sampling strategy enabled a broad group of views to be included. Different environments and disease experiences are likely to influence findings.

Nonetheless, it is clear from our findings that school closures during an influenza pandemic represent both a challenge for public health officials and a litmus test for the level of trust in public officials and government. During a public health emergency, schools that are closed act as agents of public health emergency managers. Schools therefore have a significant role to play in the public health response to an infectious disease outbreak.

## Competing interests

The authors declare that they have no competing interests.

## Authors’ contributions

ABM contributed to the design of the research and to the interpretation of data. ABM also substantially contributed to later drafts of this manuscript. RT contributed substantially to the coordination, collection, analysis and interpretation of the data. RT also contributed substantially to drafting this manuscript. JEC contributed to the collection of data and initial analysis and interpretation of the data. JEC also contributed to initial drafts of this manuscript and revision of later drafts. JMS contributed to the interpretation of data, the drafting of the manuscript and critical revision of it. HM conceptualised and designed this research and is the Principal Investigator on the NHMRC grant that funded it. HM also contributed to the interpretation of the data and drafting the manuscript. HM has approved the manuscript in its current form for publication. All authors have approved the final version of the manuscript for publication.

## Pre-publication history

The pre-publication history for this paper can be accessed here:

http://www.biomedcentral.com/1471-2458/13/344/prepub
